# Sclérodermie systémique associée à l’exposition à la silice survenant après une exposition professionnelle à la soudure à l’arc

**DOI:** 10.11604/pamj.2016.25.70.10390

**Published:** 2016-10-04

**Authors:** Zeineb Alaya, Houda Kalboussi, Walid Osman, Nader Naouar, Héla Zeglaoui, Elyès Bouajina

**Affiliations:** 1 Service de Rhumatologie, CHU Farhat-Hached, Sousse, Tunisie; 2 Service de Médecine du Travail, CHU Farhat-Hached, Sousse, Tunisie; 3 Service d’Orthopédie, CHU Sahloul, Sousse, Tunisie

**Keywords:** Sclérodermie systémique, silice cristalline, maladie professionnelle, Systemic sclerosis, silica crystalline, occupational disease

## Abstract

La sclérodermie systémique liée à l’exposition à la silice cristalline peut apparaître chez les personnes utilisant la soudure à l’arc. Une forme diffuse de sclérodermie a été diagnostiquée chez un plombier-soudeur de 57 ans, qui présentait des polyarthralgies inflammatoires, un phénomène de Raynaud, une sclérodactylie, une sclérose cutanée diffuse, des télangiectasies, une atteinte œsophagienne, une hypertension artérielle pulmonaire et une fibrose pulmonaire associées à la présence d’anticorps anti-nucléosomes. Au cours de son activité professionnelle, le patient était fréquemment exposé à des concentrations atmosphériques élevées de silice cristalline lors de la soudure à l’arc. Le diagnostic d’un syndrome d’Erasmus avec une association d’une sclérodermie systémique à une silicose pulmonaire était retenu. Une déclaration en maladie professionnelle au titre du tableau n°17 en Tunisie a été réalisée.

## Abstract (en)

Crystalline silica-associated systemic sclerosis can occur in people operating arc welding. Diffuse scleroderma was diagnosed in a 57-year old plumber-welder suffering from inflammatory polyarthralgias, Raynaud’s phenomenon, sclerodactyly, diffuse cutaneous scleroderma, telangiectasias, esophageal damage, pulmonary arterial hypertension and pulmonary fibrosis associated with the presence of anti-nucleosome antibodies. During his professional activity the patient was frequently exposed to high atmospheric concentrations of crystalline silica generated by arc-welding. The diagnosis of Erasmus syndrome associated with systemic sclerosis and pulmonary silicosis was retained. A report of work-related illness (table 17 in Tunisia) was made.

## Introduction

La sclérodermie systémique (ScS) est une maladie auto-immune rare dont l’incidence varie de 4,5 à 18,7 nouveaux cas par million d’habitants aux États-Unis et dans les pays européens [1, 2]. Elle est caractérisée par une atteinte des artérioles et des microvaisseaux, du tissu conjonctif, et par l’existence de marqueurs biologiques d’auto-immunité [3]. Les conséquences du processus pathogénique sont une oblitération vasculaire responsable de phénomènes ischémiques ainsi qu’une fibrose cutanée et viscérale [3]. Sa physiopathologie reste encore à ce jour mal élucidée. Le mécanisme de cette maladie est probablement multifactoriel: sur un terrain génétiquement prédisposé interviendraient certains facteurs exogènes [4, 5]. Parmi eux, le rôle d’une exposition à des toxiques prend une place de plus en plus importante [4]. Les facteurs environnementaux connus pour déclencher l’apparition d’une ScS sont, entre autres, la silice cristalline, les solvants organiques, les résines époxy, le chlorure de vinyle et les pesticides [3, 6]. Les travailleurs qui ont une exposition professionnelle à ces facteurs environnementaux sont surtout ceux qui travaillent dans les mines ou dans d’autres secteurs de l’industrie [4]. L’observation que nous rapportons ici détaille un cas de ScS diffuse [7] survenue chez un plombier-soudeur après une exposition professionnelle à la silice lors de la soudure à l’arc.

## Patient et observation

**Figure 1:**
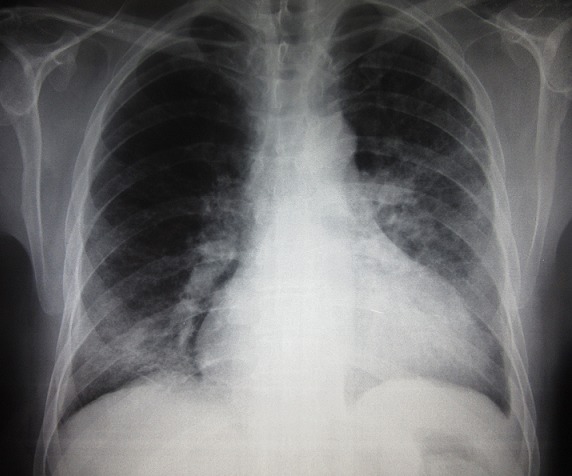
Radiographie du thorax: opacités linéaires et réticulées, micronodulaires et nodulaires diffuses, évoquant un syndrome interstitiel

**Figure 2:**
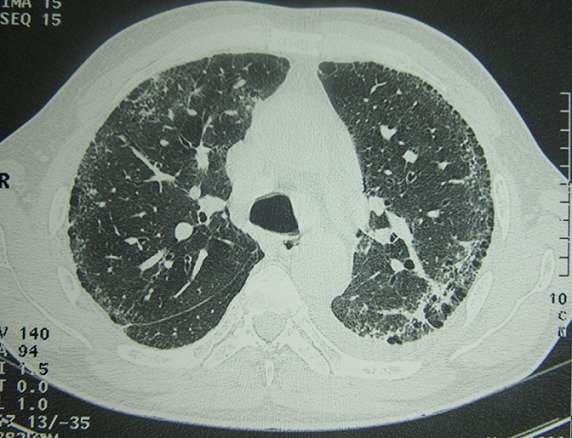
TDM thoracique: épaississement des lignes septales et non septales avec un aspect en rayon de miel associé à un foyer de condensation parenchymateux basal gauche en rapport avec une fibrose pulmonaire avec une prédominance des lésions en périphérie et aux bases

Un homme âgé de 57 ans, qui travaillait pendant 14 ans comme plombier-soudeur, était hospitalisé pour polyarthralgie inflammatoire touchant les grosses articulations évoluant depuis un an, associée à une acrosclérose rapidement extensive touchant les membres et le visage, un syndrome de Raynaud, une dysphagie avec installation depuis 4 mois d’une dyspnée d’effort stade II-III de NYHA. L’examen clinique objectivait une sclérose cutanée diffuse, un effacement des rides du visage, un nez effilé avec des télangiectasies, une limitation de l’ouverture buccale, une sclérodactylie et un syndrome de Raynaud sans ulcérations pulpaires. La mobilité des hanches et des épaules était limitée surtout sur les mouvements de rotation. L’auscultation pulmonaire trouvait des râles crépitants prédominants aux deux bases. L’auscultation cardiaque était normale et la tension artérielle était à 130/80mmHg. Le reste de l’examen clinique était sans particularités. Un syndrome inflammatoire biologique était noté. La fonction rénale était correcte. Le bilan immunologique mettait en évidence des AAN positifs de type moucheté à 1/800. Leur typage concluait à la présence d’anti-nucléosome. L’imagerie standard ostéoarticulaire était sans anomalies. La radiographie du thorax montrait des opacités linéaires et réticulées, micronodulaires et nodulaires diffuses, évoquant un syndrome interstitiel (Figure 1). La tomodensitométrie thoracique venait confirmer ce diagnostic en mettant en évidence un épaississement des lignes septales et non septales avec un aspect en rayon de miel associé à un foyer de condensation parenchymateux basal gauche en rapport avec une fibrose pulmonaire avec une prédominance des lésions en périphérie et aux bases (Figure 2). Cette atteinte respiratoire était confirmée par une gazométrie qui objectivait une hypoxie à 87,7mmHg associée à une alcalose respiratoire (pH à 7,54 et pCO2 à 32,6mmHg). L’étude fonctionnelle respiratoire révélait un trouble ventilatoire restrictif grave sans atteinte de la membrane alvéolocapillaire avec une diminution de la capacité pulmonaire totale (CPT) à 3.94L, soit < 60% et du volume expiratoire maximal en une seconde (VEMS) à 1,93L/seconde, soit 60% avec un rapport DLCO/CPT à 83%. Le test de marche de 6 minutes montrait une distance de marche anormale avec désaturation au cours de l’exercice. L’échographie cardiaque objectivait une élévation de la pression artérielle pulmonaire (PAPS=46mmHg) avec un aspect de cœur mixte ischémique et cœur pulmonaire chronique avec une fraction d’éjection du ventricule gauche à 51%. Une capillaroscopie périunguéale montrait une désorganisation avec raréfaction du lit capillaire périunguéal associé à des signes de dystrophie avec présence de signes de microangiopathie spécifique à type de mégacapillaires. Une fibroscopie digestive montrait une œsophagite grade B de Los Angeles avec bulbite ulcérée. L’examen ophtalmologique était normal. L’enquête professionnelle mettait en évidence une exposition aux particules de silice provenant de l’activité de soudure à l’arc qui remontait à 29 ans. Le diagnostic de sclérodermie systémique cutanée diffuse selon la classification de LeRoy associée à une silicose pulmonaire était retenu. Il s’agissait bien d’un syndrome d’Erasmus. Le patient était mis sous colchicine (1mg/j), oméprazole et diltiazem (120 mg/j). Une déclaration en tant que maladie professionnelle (Tableau N°17 en Tunisie) a été faite. L’évolution était marquée par l’amélioration partielle du phénomène de Raynaud. Toutefois, la gêne respiratoire persistait.

## Discussion

Plusieurs cas de connectivites spécialement de ScS secondaires à une exposition à la silice ont été rapportés [8]. En 1957, Erasmus confortait ces données en rapportant 16 cas de ScS dans un groupe de 8000 ouvriers travaillant dans des mines d’or d’Afrique du Sud; six d’entre eux avaient une silicose associée [9]. Erasmus soulignait la fréquence particulière de la ScS chez ces mineurs, comparée à celle observée dans un groupe témoin [9]. L’association d’une exposition à la silice (avec ou sans silicose) avec la ScS porte depuis le nom de « syndrome d’Erasmus » [9, 10]. En 1967, Rodnan et al. [11] confirmaient cette association en rapportant, sur 60 cas de sclérodermies masculines, une exposition prolongée à la poussière de silice chez 26 des patients (soit 43 %). Pour les auteurs, la prévalence de la ScS parmi les sujets exposés était de 17/100 000 contre respectivement six et neuf sur 100 000 chez l’homme et la femme non exposés. Pour Haustein et al. [12] dans une étude publiée en 1990, le risque de développer une sclérodermie était 25 fois plus élevé chez les sujets masculins exposés à la silice que chez ceux non exposés. Chez notre patient, le diagnostic de ScS diffuse [7] induite par la silice a été porté sur la base d’expositions répétées à la silice cristalline lors de l’activité de soudure à l’arc. Pour Magnant et al. [13], une association significative entre l’exposition à des produits toxiques, notamment la silice cristalline, et la forme diffuse de ScS avec une atteinte pulmonaire a été rapportée, ce qui a été observé chez notre patient. Les syndromes auto-immuns induits par la silice ont été expliqués par un effet de type adjuvant de la silice [14]. Les récents progrès de l’immunologie moléculaire ont permis une analyse plus détaillée des effets immunologiques de la silice. Celle-ci met en jeu des cellules immunocompétentes et aboutit à des effets qui peuvent être associés à la survenue de complications, notamment des pathologies auto-immunes, chez les personnes exposées. Des auto-anticorps contre la topo-isomérase I, la desmogléine, la caspase-8 et Fas ont été détectés dans le sérum de patients atteints de silicose [14]. Les auto-anticorps dirigés contre la caspase-8 et Fas semblent intéressants car les molécules cibles jouent un rôle clé au cours de l’apoptose des lymphocytes. Fas (CD95) est principalement exprimé sur la membrane cellulaire des lymphocytes et les auto-anticorps anti-Fas peuvent entraîner directement une apoptose des cellules portant cet antigène [14]. La découverte d’une ScS chez un patient ayant été en contact avec la silice doit faire l’objet d’une déclaration en tant que maladie professionnelle et amener idéalement à un reclassement professionnel [3, 4, 15]. Ainsi l’enquête toxique (professionnelle et non professionnelle) devant tout patient atteint de sclérodermie systémique se justifie à deux titres: la reconnaissance possible de la ScS en tant maladie professionnelle et une meilleure connaissance des toxiques impliqués dans cette pathologie [3]. Il nous parait utile, à la lumière de cette observation et pour insister sur l’importance des mesures de prévention dans les professions exposées, de souligner les risques encourus par le maniement de substances toxiques, notamment la silice. Cela s’avère indispensable dans la prévention des maladies handicapantes; en l’occurrence, la sclérodermie [16, 17].

## Conclusion

L’enquête professionnelle et extraprofessionnelle devant tout patient atteint de sclérodermie systémique se justifie à deux titres: une meilleure connaissance des toxiques impliqués dans cette pathologie et une éventuelle reconnaissance ultérieure de la sclérodermie en tant que maladie professionnelle.
